# Recombinant Myxoma Virus in European Brown Hares, 2023–2024 

**DOI:** 10.3201/eid3108.241969

**Published:** 2025-08

**Authors:** Luisa Fischer, Erwin de Bruin, Evelien Jongepier, Erik Koffeman, Patricia König, Florian Pfaff, Martin Peters, Judith M.A. van den Brand, Marin Bussi, Dominik Fischer, Valentina Caliendo, Erik Weerts, Jooske IJzer, Janina Müller, Ann-Katrin Kühling, Maren Kummerfeld, Jana Müller, Henning Petersen, Sabine Merbach, Martin Beer, Mathilde Uiterwijk, Karst de Boer, Jolianne M. Rijks

**Affiliations:** Research Center for Hunting Science and Wildlife Management, State Agency for Consumer Protection and Nutrition North Rhine-Westphalia, Bonn, Germany (L. Fischer); Dutch Wildlife Health Centre, Utrecht University, Utrecht, the Netherlands (E. de Bruin, J.M.A. van den Brand, M. Bussi, V. Caliendo, E. Weerts, J. IJzer, J.M. Rijks); Royal Dutch Hunters’ Association, Amersfoort, the Netherlands (E. Jongepier); Fauna Management Unit Gelderland, Arnhem, the Netherlands (E. Koffeman); Friedrich-Loeffler-Institut, Greifswald-Insel Riems, Germany (P. König, F. Pfaff, M. Beer); Chemical and Veterinary Investigation Office Westphalia, Arnsberg, Germany (M. Peters, S. Merbach); Der Grüne Zoo Wuppertal, Wuppertal, Germany (D. Fischer); Chemical and Veterinary Investigation Office Rhein-Ruhr-Wupper, Krefeld, Germany (Janina Müller, A.-K. Kühling); Chemical and Veterinary Investigation Office Münsterland-Emscher-Lippe, Münster, Germany (M. Kummerfeld); Chemical and Veterinary Investigation Office Ostwestfalen-Lippe, Detmold, Germany (Jana Müller, H. Petersen); Centre for Monitoring of Vectors, Netherlands Food and Consumer Product Safety Authority, Wageningen, the Netherlands (M. Uiterwijk, K. de Boer)

**Keywords:** myxoma virus, viruses, hare, wild animals, Lagomorpha, myxomatosis, poxviridae infections, Germany, the Netherlands

## Abstract

Recombinant myxoma virus has emerged in European brown hares (*Lepus europaeus*), causing increased deaths associated with swollen eyelids, head edema, and dermatitis at face, legs, and perineum. Introduction may date back as far as September 2020. As of August 2024, the disease is spreading radially from the Germany–Netherlands border area.

In August 2024, reports of sick and dead European brown hares (*Lepus europaeus*) showing swollen eyelids, edema of head and ears, and dermatitis of face, legs, and perineum increased in the Germany–Netherlands border area of the federal state of North Rhine-Westphalia, Germany, and the provinces of Overijssel and Gelderland, the Netherlands (Figure 1, panel A). The clinical picture resembled myxomatosis, a disease caused by myxoma virus (MYXV; *Leporipoxvirus myxoma*, family *Poxviridae*). In 2023, a total of 4 European brown hares with similar lesions had been submitted for pathologic investigation in 2 adjacent North-Rhine Westphalia municipalities, but those cases were then thought to be sporadic MYXV cases, as reported elsewhere (*1*,*2*). 

**Figure 1:**
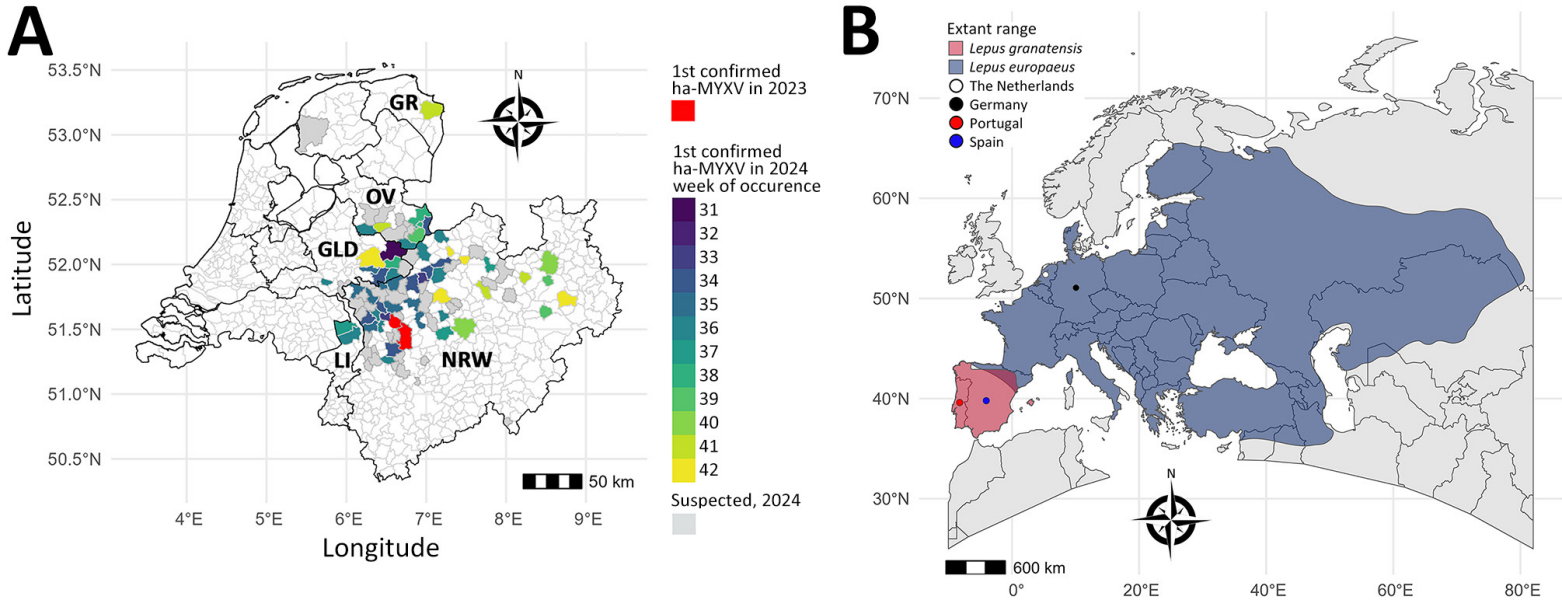
Location of an outbreak of ha-MYXV during 2024 in European brown hares (*Lepus europaeus*) in the border area of the Netherlands and Germany compared with ranges of the hares and the Iberian hare (*Lepus granatensis*). A) Municipalities and provinces in the Netherlands and the Germany federal state of North-Rhine Westphalia, showing the presumed epicenter of the outbreak (municipalities in which ha-MYXV occurred in 2023), as well as the spatiotemporal development of the outbreak in European brown hares during August 1–October 20, 2024. Confirmed ha-MYXV cases occurred in the Netherlands provinces Limburg, Gelderland, Overijssel, and Groningen and in North-Rhine Westphalia. Municipalities without laboratory-confirmed ha-MYXV cases during the study period, but where ha-MYXV-infection in hares was suspected on the basis of pathology, electron microscopy, or photographs of affected hares provide an indication of the probable area of virus presence, are indicated. Map created in R version 4.4.1 (The R Project for Statistical Computing, https://www.r-project.org). B) Extant ranges of both hare species. The outbreak area in northwest Europe is in the western part of the extant range of the European brown hare and far from the extant range of the Iberian hare, in which ha-MYXV was discovered in 2018, causing disease in both Spain and Portugal. Source of extant range shapes: International Union for Conservation of Nature. ha-MYXV, hare-adapted natural recombinant myxoma virus.

In Europe, MYXV was intentionally released in the 1950s as biological control for the European rabbit (*Oryctolagus cuniculi*), causing massive disease (*3*). Although outbreaks were less frequent and severe by time because virulence decreased and immunity increased in the rabbit population (*4*), a hare-adapted natural recombinant MYXV (ha-MYXV) emerged in 2018 in Iberian hares (*Lepus granatensis*) on the Iberian Peninsula (*5*,*6*) (Figure 1, panel B). Before then, mass deaths associated with MYXV were not known to occur in European brown hares. We investigated the 2024 outbreak in the Germany–Netherlands border area.

## The Study

Wild lagomorphs were submitted from Germany and the Netherlands for investigation during August 1–October 20, 2024. Lagomorphs in this study were shot or found dead with myxomatosis-suspected lesions and submitted for postmortem examination; no animal was killed for the study. We performed pathological examination on 193 myxomatosis-like hares (159 from Germany, 26 from the Netherlands) and wild rabbits (6 from Germany, 2 from the Netherlands), mostly adult animals of both sexes. Body condition varied; 41 (21.2%) animals were cachectic, and of those, 39 (30 from Germany, 9 from the Netherlands) were hares and 2 (1 from each country) were rabbits. 

**Figure 2:**
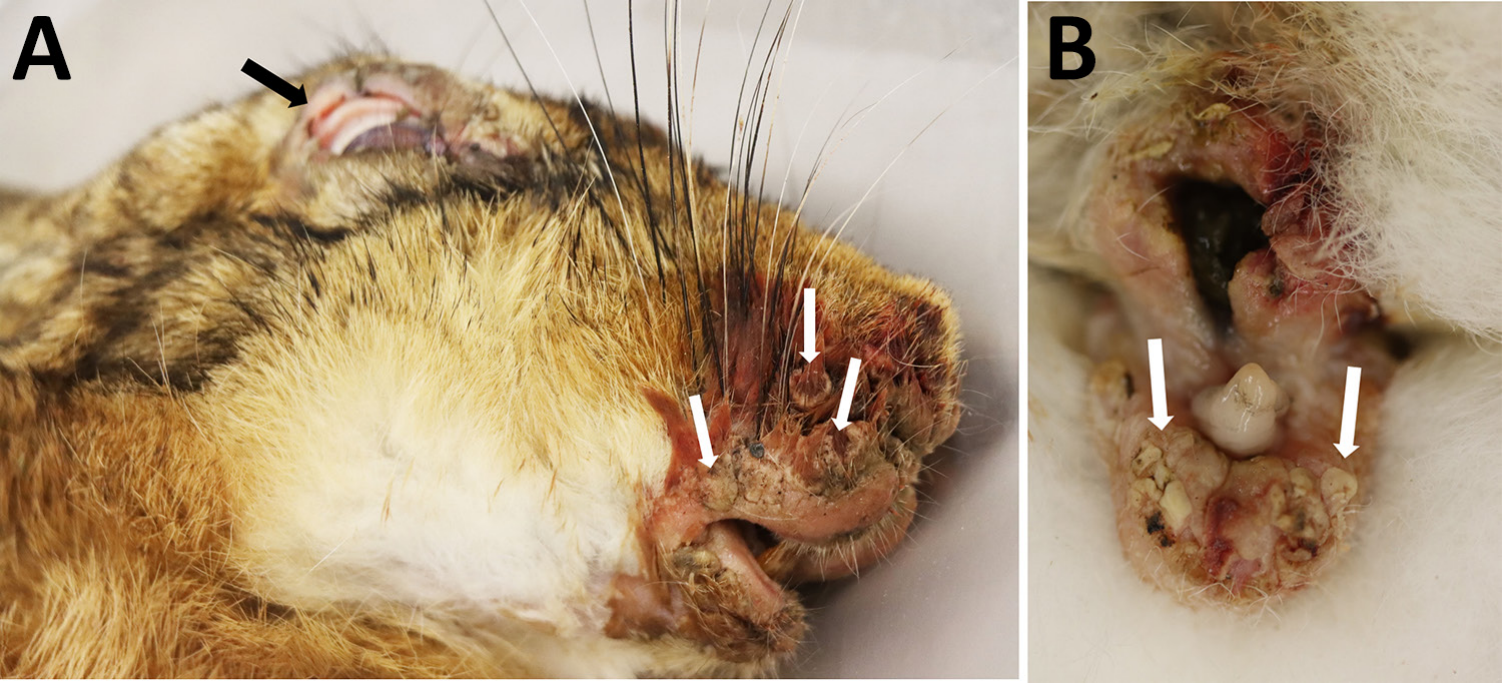
European brown hare (*Lepus europaeus*) with myxomatosis caused by a hare-adapted natural recombinant myxoma virus during a 2024 outbreak in the border area of the Netherlands and Germany. A) Conjunctivitis (black arrow) and nodular skin proliferations at the lips and nose (white arrows). B) Inflammatory swelling of male genital mucous membranes with ulcerations (white arrows).

The conjunctivae and skin surrounding the eyes, nose, ears, perineum, and legs were thickened with secondary inflammation (Figure 2, panels A, B), orthokeratotic hyperkeratosis, acanthosis, intracorneal pustules, ulceration, and crust formation. Vacuolated keratinocytes, often with regular intracytoplasmic eosinophilic inclusion bodies, exocytosis by heterophilic granulocytes, and apoptosis were prominent in the intact epithelium, including that of the adnexa. Proliferation of pleomorphic mesenchymal cells (myxoma cells) with moderate anisocytosis and anisokaryosis, embedded in myxoid stroma (Appendix 1 Figure 1, panel A), was visible in the surrounding stroma, often accompanied by extensive infiltration of heterophilic granulocytes. Myxoma cells inconsistently contained prominent intracytoplasmic inclusion bodies and fewer amphophilic intranuclear inclusion bodies. The lesions were consistent with myxomatosis; electron microscopic findings further supported that determination (Appendix 1 Figure 1, panel B). In some cases, we diagnosed secondary bacterial infections of the lesions, as well as co-infections (Appendix 2 Table 1).

**Figure 3:**
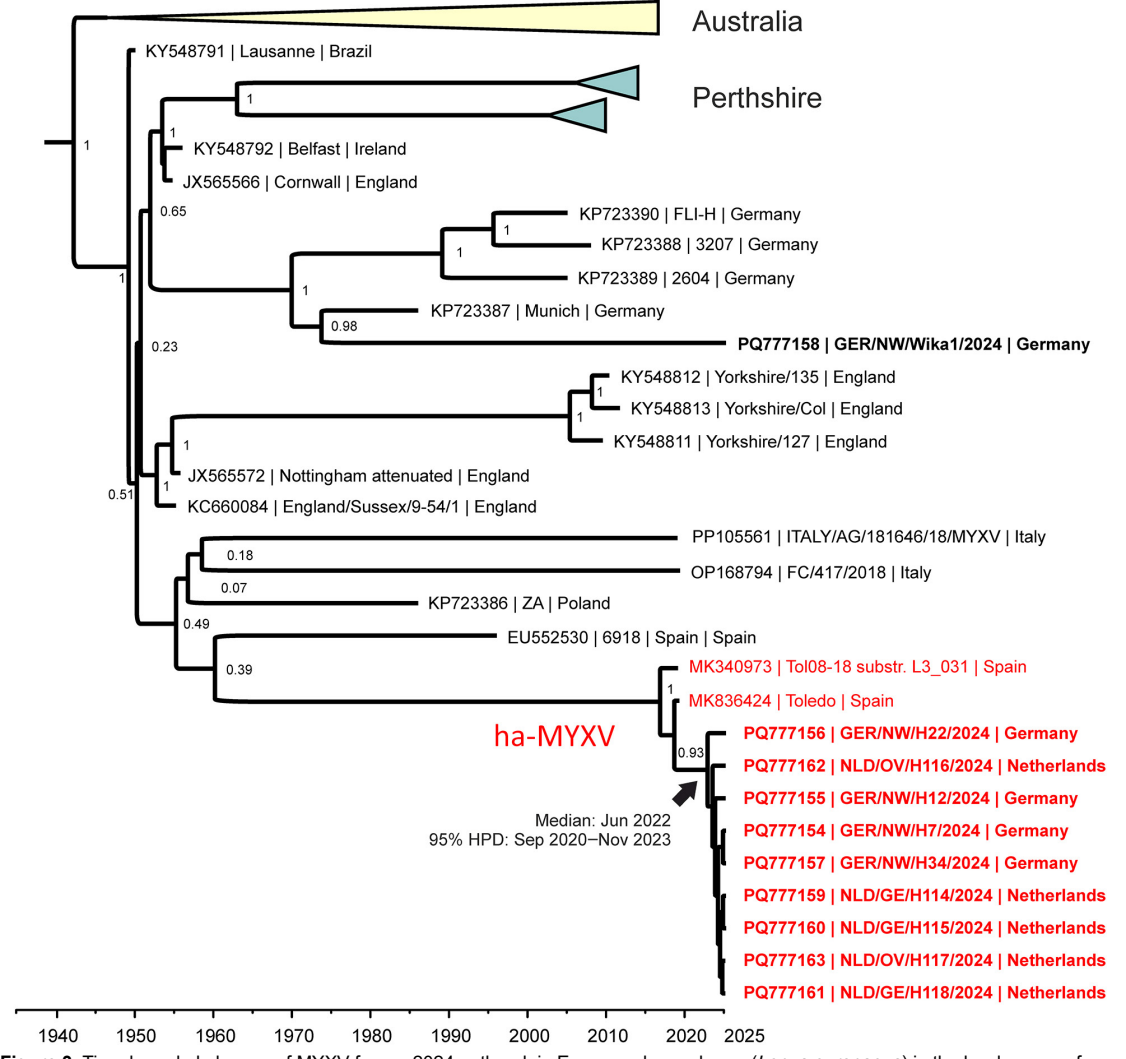
Time-based phylogeny of MYXV from a 2024 outbreak in European brown hares (*Lepus europaeus*) in the border area of the Netherlands and Germany and reference sequences. Ten full-length MYXV sequences from the outbreak were aligned to 114 available full-length MYXV reference genome sequences from GenBank and used for time-based phylogenetic analyses with BEAST version 1.10.4 (https://github.com/beast-dev/beast-mcmc/releases/tag/v1.10.4). Red indicates isolates belonging to ha-MYXV; bold text indicates sequences from this study. Branch labels represent statistical support values; values closer to 1 indicate stronger support. GenBank accession numbers are shown. ha-MYXV, hare-adapted natural recombinant MYXV; HPD, highest posterior density; MYXV, myxoma virus.

We confirmed myxomatosis by MYXV-specific real-time quantitative PCR (qPCR) analyses of eyelid, skin, or lung samples in 104 hares (78 from Germany, 26 from the Netherlands) and 8 rabbits (6 from Germany, 2 from the Netherlands) (Appendix 2 Table 2). We further categorized MYXV-positive samples into classical and ha-MYXV by a second strain-specific qPCR test (*7*). In total, all 104 hares and half (4/8) of the wild rabbits tested positive for the recombinant ha-MYXV; the remaining rabbits tested positive for classical MYXV. No case of double infection was detected. We performed full-genome sequencing on virus cultured from eyelid samples of 9 hares (4 from Germany, 5 from the Netherlands, all ha-MYXV) and 1 wild rabbit (classical MYXV) to confirm PCR results, enable comparisons with other MYXV, and give insight into the evolutionary history of the virus. We prepared DNA libraries and sequenced on the long-read sequencing platform PromethION (Oxford Nanopore Technologies, https://www.nanoporetech.com) (Appendix 1). We trimmed and de novo assembled the raw reads and aligned the resulting MYXV genome sequences with all available MYXV references. We submitted annotated MYXV genome sequences to the International Nucleotide Sequence Database Collaboration (https://www.insdc.org; accession nos. PQ777154–63). A time-structured phylogenetic analysis indicated that the MYXV genomes from hares from the 2024 outbreak have evolved from the same lineage of ha-MYXV that caused mass deaths in Iberian hares (Figure 3). Consistent with the qPCR results, the sequence from the wild rabbit clustered with classical MYXV strains from Germany. Time-aware phylogenetic analysis estimated that the most recent common ancestor of the sequenced ha-MYXV genomes could have emerged as early as September 2020; mean estimated date was June 2022 (95% highest posterior density September 2020–November 2023) (Figure 3).

We retrieved formalin-fixed paraffin-embedded (FFPE) samples from 5 hares found dead during October 2023–April 2024 in the municipalities of Rheinberg, Germany (n = 4) and Duisburg, Germany (n = 1) for retrospective virological examination. PCR results confirmed ha-MYXV infection, demonstrating the presence of pathogen in 2023 (Figure 1, panel A; Appendix 2 Tables 1, 2).

For further insight into the outbreak’s probable epicenter and the pattern of spread, we identified municipalities with qPCR-confirmed ha-MYXV cases in 2024 and plotted them by week of first detection. For an overview of the probable area affected, we identified municipalities without confirmed but with suspected cases. We classified hares as suspected cases if pathology results suggested myxomatosis or, for reported hares not submitted for examination, if photographs showed myxomatosis-like lesions (Appendix 2 Table 1). The map suggested a radial and northward spread (Figure 1, panel A). The increased occurrence of ha-MYXV in hares was assumed to be associated with abundance of biting insects such as mosquitoes (Appendix 1 Figure 2), similar to transmission of classical MYXV and as assumed in previous studies (*8*). However, ha-MYXV was not detected via qPCR in 28 mosquitoes collected at 3 different locations in Germany that had confirmed ha-MYXV cases (Appendix 1 Figure 3).

To assess the immediate effect on the hare population, we used autumn hare counts conducted by hunters using thermal imaging from the Province of Gelderland, Netherlands. We compared the number of hares counted in October 2024 with the average count in the 3 preceding years. The results showed a population decline in municipalities with confirmed and suspected cases of ha-MYXV, compared with municipalities without reports of the pathogen (W = 61, p<0.001 by Wilcoxon signed-rank test) (Appendix 1 Figure 4; Appendix 2 Table 3).

## Conclusions

This study demonstrated that ha-MYXV infection caused death in European brown hares. This hare species has a much wider distribution than the Iberian hare; its extant range overlaps with other native hare species in Europe, such as the mountain hare (*Lepus timidus*) and the vulnerable Corsican hare (*Lepus corsicanus*). Our findings also confirm previous results of ha-MYXV infection and death in European rabbits (*9*); however, the effect of this additional hare-adapted variant on the rabbit population is yet unknown. The pattern of disease spread in hares seems to be radial and northward. The outbreak occurrence in late summer suggests transmission by arthropods (*10*). Collectively, those results indicate that ha-MYXV could spread widely in lagomorphs in Europe and possibly beyond. The appearance of ha-MYXV in a central location in northwest Europe with a radial spread, far away from its origin at the Iberian Peninsula, is most likely the result of pathogen introduction via anthropogenic transport of contaminated fomites, vectors, or infected live or dead leporids. Our results indicated that ha-MYXV was already present in the outbreak area in 2023, and time-aware phylogenetic analyses suggest that introduction may date back as far as September 2020. Despite variation, municipalities with diseased hares showed on average a stronger decline in hare counts than those in which no ha-MYXV was reported. Those findings suggest that, at least in the short term, ha-MYXV affects the hare population in this region, and the disease is spreading.

Appendix 1 Additional information from study of recombinant myxoma virus in European brown hares, 2023–2024.

Appendix 2 Data from study of recombinant myxoma virus in European brown hares, 2023–2024.
