# Supplementary material for: Recombinant Myxoma Virus in European Brown Hares, 2023–2024
Source: Emerg Infect Dis. 2025 Aug;31(8):1608–12. doi: 10.3201/eid3108.241969 (PMC12309757; doi:10.3201/eid3108.241969)
Supplement: Appendix 1 — Additional information from study of recombinant myxoma virus in European brown hares, 2023–2024. [file 24-1969-Techapp-s1.pdf]

Article DOI: https://doi.org/10.3201/eid3108.241969

EID cannot ensure accessibility for supplementary materials supplied by authors. Readers who have difficulty accessing supplementary content should contact the authors for assistance.

Recombinant Myxoma Virus in European Brown Hares, 2023–2024

Appendix 1

Material and Methods

Pathology

Hares shot or found dead with myxomatosis-suspected lesions were collected in the field and admitted to the veterinary diagnostic offices in North Rhine-Westphalia, Germany, and the Dutch Wildlife Health Centre (DWHC), Utrecht University (Utrecht, The Netherlands) for monitoring and surveillance purposes. The hares were necropsied according to standard protocols. Tissues were collected and stored at −20°C or −80°C for virology and in 10% neutral buffered formalin for histopathology. After fixation in formalin, the tissues were paraffin embedded and after processing, 3 µm sections were stained with hematoxylin and eosin (H&E) for examination by light microscopy.

Electron Microscopy

Ultrastructural examination was performed with negative staining according to the recommendation of the Robert Koch Institute, Germany. Briefly, the unfixed skin lesions were scarified and placed against formvar-filmed electron microscopy copper grids to adsorb virus particles. These were negatively contrasted with 3% (w/v) phosphotungstic acid for 30 sec and examined with an LEO0906 Electron microscope (Zeiss, Germany).

Nucleic Acid Extraction

Individual or pooled skin samples were homogenized on a Tissue LyserII (Qiagen, Hilden, Germany) at 30 Hz for 2.5 min. Homogenates were cleared by centrifugation (12,850 × g, 2 min) and 200 µl of supernatants were subjected to manual DNA extraction (High Pure PCR Template Preparation Kit; Roche Diagnostics, Mannheim, Germany) or to automated nucleic acid extraction using NucleoMag VET (Macherey-Nagel, Düren, Germany), according to the kit

instructions, on a KingFisher Flex purification system (Thermo Fisher Scientific, Waltham, USA). DNA extraction from the formalin-fixed paraffin-embedded samples was performed with the DNeasy® Blood & Tissue Kit (Qiagen, Hilden, Germany) following the manufacturer's protocol for paraffin-embedded tissue.

PCR Amplification

For MYXV diagnosis and differentiation the published quadruplex real time PCR was modified and divided into two separate duplex reactions (*1*). In a first step, presence of myxomatosis virus was confirmed and DNA quality and possible inhibition were simultaneously monitored in a total volume of 15 µl, using 7.5 µl QuantiTect Multiplex PCR noRox 2×Mastermix (Qiagen, Hilden, Germany), 660 nM of each MYXV *m000.5L/R* primer, 160 nM *m000.5L/R* probe and 160 nM of *β-actin* primers with 83 nM of the actin probe and 5 µL template DNA (*2*). Cycling conditions were set to 10 min at 95°C initial denaturation followed by 42 cycles of denaturation (30 sec, 95°C), annealing (30 sec, 50°C) and elongation (30 sec, 60°C).

The QuantiTect Multiplex PCR noRox mastermix (Qiagen, Hilden, Germany) was also selected for the differentiation of the causative myxoma virus. A DNA volume of 5 µl was analyzed in a final reaction volume of 25 µl. The final primer-probe concentrations for the ha-MYXV assay were adjusted to 303 nM of each MYXV *m060L* primer and 57 nM of the corresponding probe. Classic myxomatosis virus was detected with the *m009L* primer (200 nM each) and probe (38 nM) set. Cycling conditions for the type specific duplex assay were accordingly composed of initial denaturation for 10 min at 95°C and 42 PCR cycles of denaturation (20 sec, 95°C), annealing (30 sec, 60°C) and amplification (30 sec, 72°C).

Amplification was done on a CFX96 PCR cycler (BioRad Laboratories, München, Germany), and real-time PCR results were analyzed using the CFX Maestro software Version 4.1 (BioRad Laboratories). A negative cut off for the MYXV *m009* reaction was set to cq>35 (cycle of quantification), corresponding for all other reactions to cq>42.

A published real time PCR protocol (*3*) was modified for the detection of glycoprotein B (gB) sequences of the leporid gammaherpesvirus 5 (LeHV-5). Our sample panels were composed by skin samples from different body sites. Preferred parenchyma for the detection of the LeHV-5 (lungs, liver, spleen) were not available. LeHV-5 gB specific amplification was

combined with an amplification control reaction targeting the cellular β-actin gene. We used the QuantiTect Multiplex PCR noRox PCR kit (Qiagen, Hilden, Germany) with primer concentrations of 800 nM of each gB primer and 200 nM gB probe as well as 160 nM β-actin primers each and 83 nM β-actin probe in a final volume of 25 µl including 5µl of template DNA. Cycling was performed after initial denaturation for 10 min at 96°C by 42 cycles of denaturation at 96°C for 20 s, annealing at 60°C for 30 s and extension at 72°C for 30 s. All primers and probes were manufactured by Metabion, Martinsried, Germany.

Virus Isolation

Isolation of infectious myxoma virus was conducted in rabbit kidney RK13 cells (Collection of Cell Lines in Veterinary Medicine CCLV-RIE 0109, FLI) cultured in minimal essential medium (MEM; Corning, NY, USA) supplemented with 10% fetal bovine serum FBS and penicillin–streptomycin (Pan Biotech, Aidenbach, Germany; 10 U ml^{-1} and 100 µg ml^{-1}, respectively). Cells were cultured in 12 well plates and inoculated 1 day post plating with 200 µl of tissue sample homogenates cleared for 3 min at 20,000 × g. After incubation at 37°C for 1 hours (adsorption) the inoculum was removed, cultures were washed twice with complete medium and replenished with 1.5 ml complete growth medium (MEM 10% FBS). Cells were monitored daily for the development of a cytopathic effect (CPE), including cell swelling, plaque formation or formation of syncytia and cell lysis. When prominent CPE was observed, infected cultures were harvested and stored at –70°C.

DNA Preparation for Genome Sequencing

RK13 cells were grown on six-well plates in 3 ml complete growth medium and infected after 1 day post plating with 400 µl of lysates from the initial cultures. Supernatants were discarded and cells were harvested when CPE was observed in an overall of 80%–90% of the cells. DNA was subjected to RNA digestion and prepared with the Puregene® Core Kit A according to the manufacturer’s instructions (Qiagen, Hilden, Germany). Samples quality was checked in both MYXV real-time assays and DNA was quantified on a NanoPhotometer® N60 spectrophotometer (Implen, München, Germany).

DNA Sequencing and De Novo Assembly

Sequencing libraries were prepared from extracted DNA using the Rapid Barcoding Kit SQK-RBK114.24 (Oxford Nanopore Technologies) according to the manufacturer's protocol.

The prepared libraries were loaded onto R10.4.1 flow cells on an Oxford Nanopore PromethION instrument configured with E8.2 sequencing chemistry. Sequencing was performed at a base calling rate of 400 bases per second. Raw sequencing data were basecalled in super accuracy mode (sup) using Guppy basecaller version 4.3.0 (Oxford Nanopore Technologies).

Raw sequencing reads were subsequently processed using a range of bioinformatics tools to ensure high quality data for downstream analysis. Barcode trimming was performed using Porechop (v0.2.4), which removed sequencing adapters and demultiplexed reads. Low quality reads were then filtered out using Filtlong v0.2.1, retaining only sequences longer than 200 bases with an average quality score greater than 10. High-quality reads were then mapped to the MYXV Lausanne reference strain (Genbank accession number KY548791) using Minimap2 (v2.28-r1209) and mapping reads were assembled de novo using Flye (v2.9.5).

Phylogenetic Analysis

The generated full-length MYXV sequences were aligned to 114 available full-length reference genome sequences from GenBank using MAFFT (v7.490). The alignment region representing the left terminal repeat and the entire ~2.8kb recombinant insert of the ha-MYXY strains was removed (and relevant columns of the classic MYXV) to avoid redundancy and misalignment, respectively. Phylogenetic analyses were performed using BEAST and BEAUti software (v1.10.4). Before the final analysis, we evaluated different combinations of substitution models ("GTR+G4+I" and "HKY+G4+I"), clock models ("Uncorrelated relaxed clock" and "Strict clock") and tree priors ("GMRF Bayesian Skyride" and "Constant size") using a MCMC chain of each 100,000,000 steps and the stepping stone and path sampling methods (path steps: 100; chain length: 1,000,000). Based on the log marginal likelihood values, we select the best combination as the phylogenetic model for the final analysis.

Finally, a GMRF Bayesian skyride model with time-dependent smoothing and a randomized starting tree was used. Population sizes were parameterised in log units and a gamma prior was applied to the precision parameter. A log-normal uncorrelated relaxed molecular clock model was used and the mean rate prior was set to 1.0×10^{-5} substitutions/site/year in accordance with Kerr et al. (*4*). The substitution model was GTR with gamma-distributed rate heterogeneity (four categories) and a proportion of invariant sites. MCMC sampling was performed for 100,000,000 steps, with logs recorded at regular intervals of

1,000 steps. Operators were automatically optimised and outputs included parameter estimates, tree files and population size trajectories.

Key statistical operators and traces were checked in the MCMC Trace Analysis Tool (v1.7.2). Specifically, the effective sample size of the root age, coalescent likelihood and tree likelihood were 734, 2983 and 6274, respectively. A maximum-clade credibility tree was generated using TreeAnnotator (v1.10.4) with 1,000,000 burn-in states and subsequently visualized using FigTree (v1.4.5_pre).

Mosquito Count Data from the Netherlands

Longitudinal vector monitoring is performed by the Centre for Monitoring Vectors (CMV) of the Netherlands Food and Consumer Product safety Authority (NVWA). Each year CMV/NVWA places mosquito traps across the Netherlands, and uses the results for insight into spatio-temporal distribution of mosquitos (https://www.nvwa.nl/onderwerpen/muggen-knutten-en-teken/rol-nvwa/monitoring).

Mosquito Collection in North Rhine-Westphalia

Box gravid traps were constructed and used according to Fynmore et al. (*5*) based on the design of the Reiter-Cummings gravid traps. Briefly, traps composed of a stackable black plastic box as lower tray (40 × 30 × 22 cm) and a black, 17 × 37 × 19 cm sized, plastic LUX Tools toolbox (Emil Lux GmbH & Co. KG, Wermelskirchen, Germany) as main trap body were used. The lower tray was filled with 5 L hay infusion as liquid attractant for the mosquitos (*5*). Via a 15 cm long and 7.5 cm in diameter wide piece of polypropylene pipe (Marley HT Pipe DN 75, Marley Deutschland GmbH, Wunstorf, Germany) air was drawn from the lower tray, ≈4 cm over the water surface, inside the toolbox using a small, battery run, axial cooling fan (LogiLink DC Brushless Fan, Model Fan101, 2direct GmbH, Schalksmühle, Germany). As the pipe passed the air into a self-made collection chamber before being expelled from the side of the toolbox, mosquitoes were transported with the air stream and trapped in the collection chamber until manually removed.

Mosquito traps were set in three locations in North Rhine-Westphalia in the period September-October 2024 (Kleve (September 2024 and October 2024), Veen (16 October 2024), and Wesel (September 2024 and 16 October 2024)). Sealed collection chambers were frozen for 2 hours to facilitate removal of frozen mosquitos from the collection chambers to petri dishes.

Species were determined with a stereo microscope using the keys provided in Becker et al., 2020 (*6*). They were handled per individual using tweezers and a dissecting needle. Both were sterilised with 70 percent ethanol between individuals as to not get cross contamination. In total 25 female and 3 male mosquitos of 6 species were obtained.

Mosquito Testing for MYXV

Mosquitos were individually homogenized using Lyzing matrix D beads (MP Biomedicals, Eschwege, Germany) and a Magnalyzer (Roche Diagnostics, Rotkreuz, Switzerland), for 6500 rpm, 30 seconds in PBS. DNA extraction was performed using the DNeasy® Blood & Tissue Kit (Qiagen, Hilden, Germany) following the manufacturer's protocol. Detection of MYXV and inhibition control was done using the MYXV m000.5L/R and β-actin PCR as described before for hares.

Impact Assessment

Night-time autumn hare counts are conducted yearly in October / November using thermal imaging. In total, 127 areas of 143.5 (median) hectare in size (IQR = 105.6 - 229.2) were counted by hunters in the Dutch province of Gelderland. For each area, the proportional change in hare counts between 2024 (as counted on 5 October 2024) and the average over the period 2021–2023 was computed, Z-score transformed to account for potentially confounding factors and averaged across Game Management Units (GMU). To obtain Z-scores per municipality, the proportional overlap in area between GMU's and municipalities was determined based on which a weighted average was computed (Appendix 2 Table 3). The Z-score per municipality was compared between municipalities with confirmed or suspected cases of ha-MYXV and those where the virus was not yet reported using a Wilcoxon signed-rank test.

References

1. Abade dos Santos FA, Carvalho CL, Parra F, Dalton KP, Peleteiro MC, Duarte MD. A quadruplex qPCR for detection and differentiation of classic and natural recombinant myxoma virus strains of leporids. Int J Mol Sci. 2021;22:12052. PubMed https://doi.org/10.3390/ijms222112052

2. Toussaint JF, Sailleau C, Breard E, Zientara S, De Clercq K. Bluetongue virus detection by two real-time RT-qPCRs targeting two different genomic segments. J Virol Methods. 2007;140:115–23. PubMed https://doi.org/10.1016/j.jviromet.2006.11.007

3. Abade dos Santos FA, Carvalho CL, Peleteiro MC, Parra F, Duarte MD. A versatile qPCR for diagnosis of leporid gammaherpesvirus 5 using Evagreen® or Taqman® technologies. Viruses. 2021;13:715. PubMed https://doi.org/10.3390/v13040715

4. Kerr PJ, Ghedin E, DePasse JV, Fitch A, Cattadori IM, Hudson PJ, et al. Evolutionary history and attenuation of myxoma virus on two continents. PLoS Pathog. 2012;8:e1002950. PubMed https://doi.org/10.1371/journal.ppat.1002950

5. Fynmore N, Lühken R, Maisch H, Risch T, Merz S, Kliemke K, et al. Rapid assessment of West Nile virus circulation in a German zoo based on honey-baited FTA cards in combination with box gravid traps. Parasit Vectors. 2021;14:449. PubMed https://doi.org/10.1186/s13071-021-04951-8

6. Becker N, Petrić D, Zgomba M, Boase C, Madon MB, Dahl C, et al. Mosquitoes: identification, ecology and control. Cham (Switzerland): Springer Nature; 2020. https://doi.org/10.1007/978-3-030-11623-1.

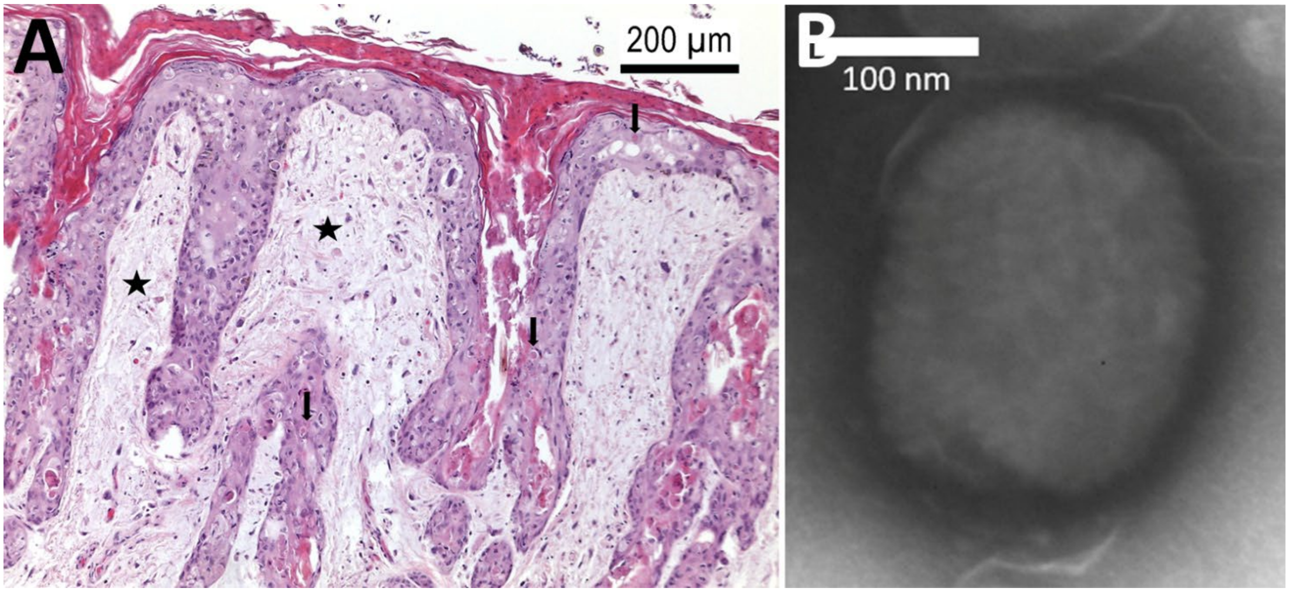

Appendix 1 Figure 1. A) Histological section of the haired skin at the lips (HE stain): epidermal hyperplasia with orthokeratotic hyperkeratosis, epithelial ballooning degeneration, intraepithelial eosinophilic intracytoplasmatic viral inclusions (black arrows), severe myxedema in the superficial dermis (star). B) negative staining of a brick-shaped leporipox virus particle with characteristic outer surface structure from within the lesion.

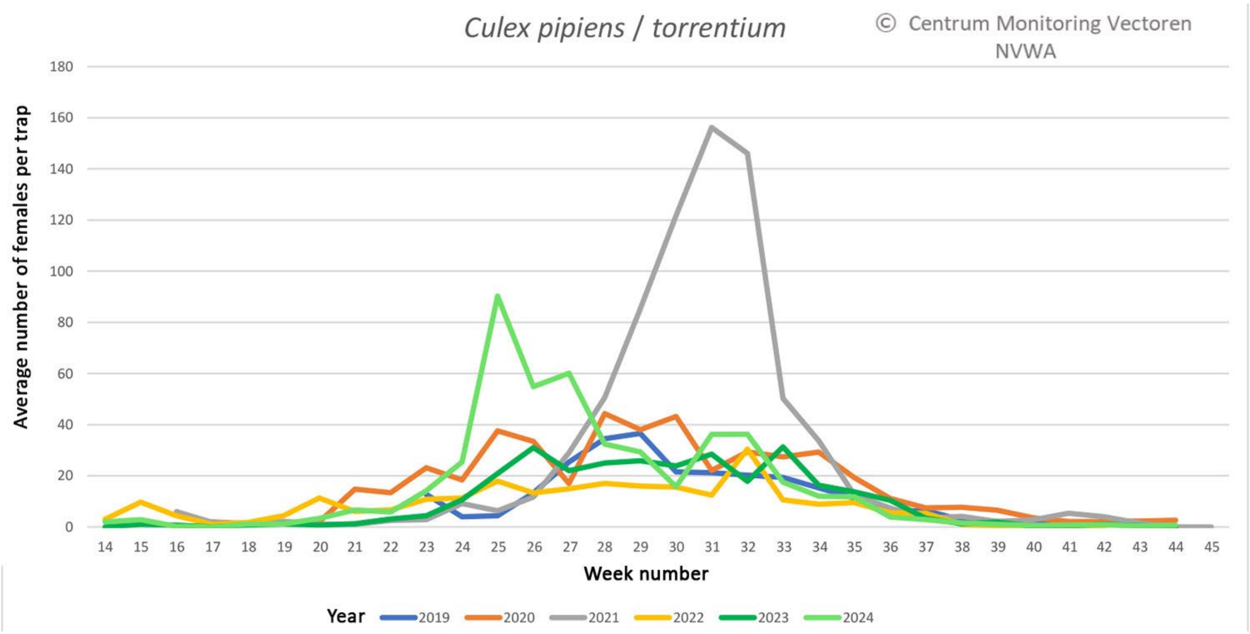

Appendix 1 Figure 2. Mosquito count data from the Netherlands (2019-2024) indicating the average number of female *Culex pipiens/torrentium* mosquitoes per trap (mosquito abundance).

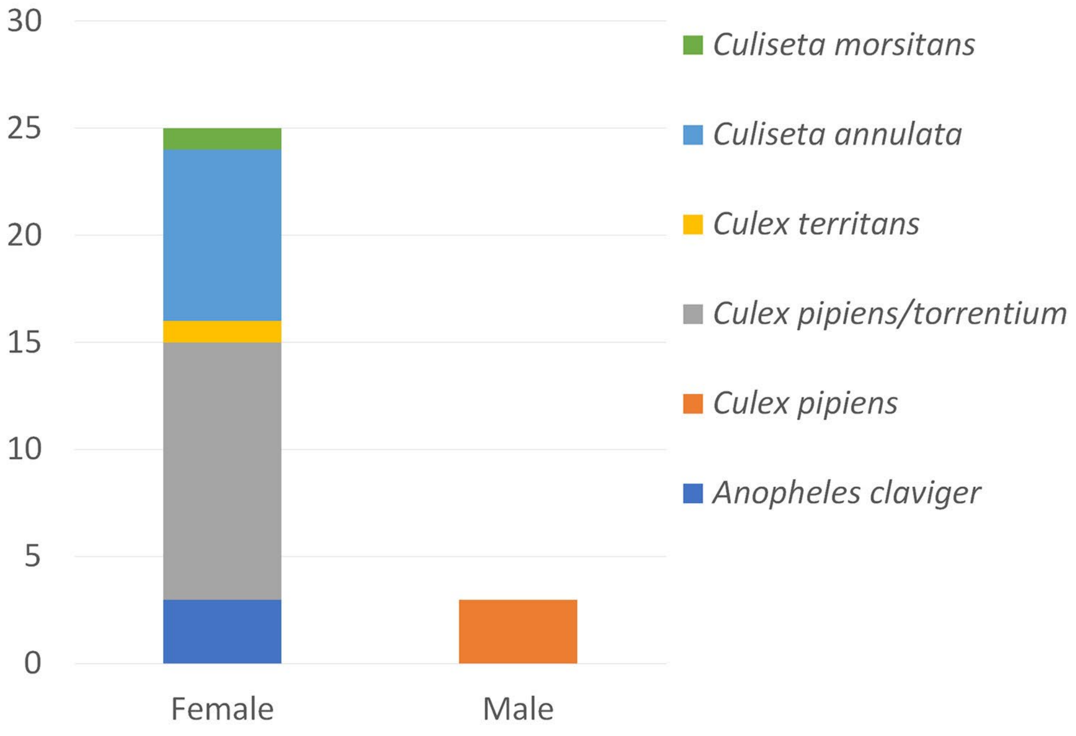

Appendix 1 Figure 3. Differentiated species and sex of mosquitoes trapped in Germany for ha-MYXV testing by qPCR.

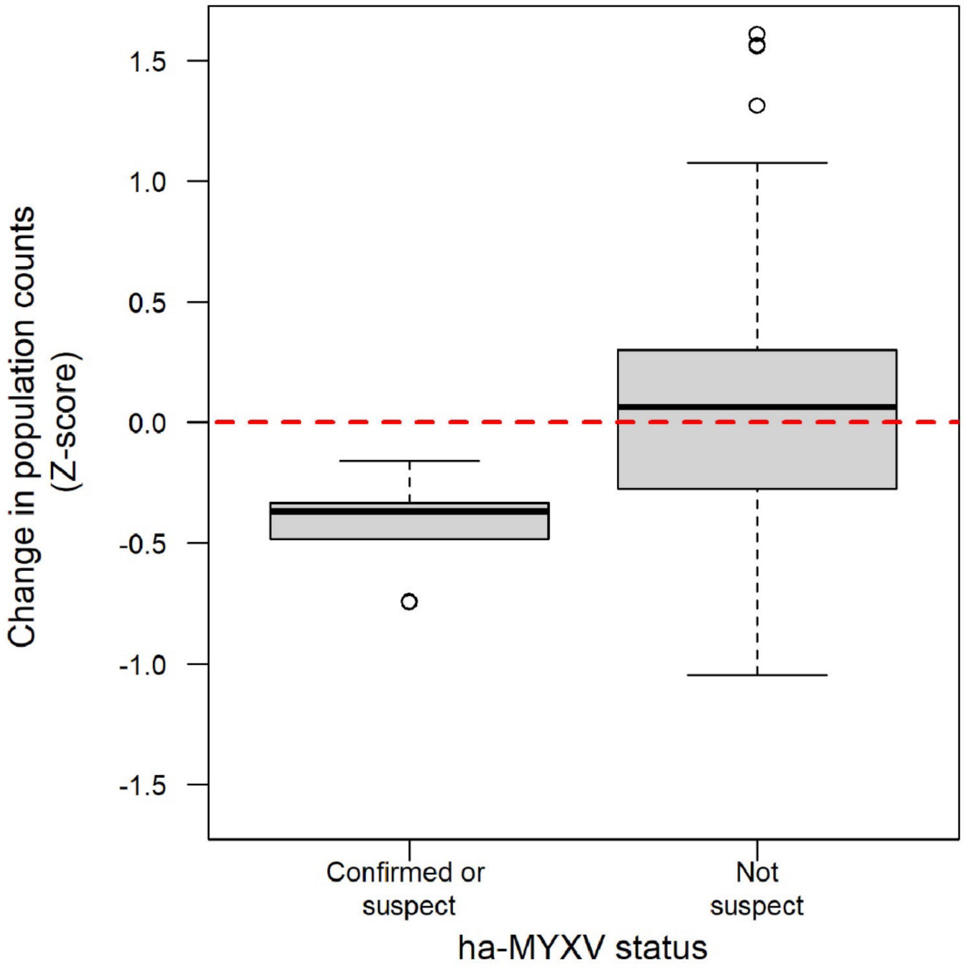

Appendix 1 Figure 4. Assessment of the immediate impact on the hare population by comparison of average autumn hare counts of the last 3 years in the Province of Gelderland, NL including municipalities with confirmed and suspected cases of ha-MYXV, compared to municipalities without reports of the pathogen.
